# Implication of scavenger receptors in the interactions between diesel exhaust particles and immature or mature dendritic cells

**DOI:** 10.1186/1743-8977-6-9

**Published:** 2009-03-13

**Authors:** Solenne Taront, Audrey Dieudonné, Simon Blanchard, Pascale Jeannin, Philippe Lassalle, Yves Delneste, Philippe Gosset

**Affiliations:** 1 INSERM, U774, Lille, F-59019, France; Institut Pasteur de Lille, Lille, F-59019, France; Univ Lille II, Lille, F-59000 France; 2 INSERM U892, Centre de Recherche sur le Cancer Nantes Angers, Angers F-49933, France; University of Angers, Angers, France; 3 Immunology and Allergology department, University Hospital of Angers, Angers, 49933, France

## Abstract

**Background:**

The exposure to pollutants such as diesel exhaust particles (DEP) is associated with an increased incidence of respiratory diseases. However, the mechanisms by which DEP have an effect on human health are not completely understood. In addition to their action on macrophages and airway epithelial cells, DEP also modulate the functions of dendritic cells (DC). These professional antigen-presenting cells are able to discriminate unmodified self from non-self thanks to pattern recognition receptors such as the Toll like Receptors (TLR) and Scavenger Receptors (SR). SR were originally identified by their ability to bind and internalize modified lipoproteins and microorganisms but also particles and TLR agonists. In this study, we assessed the implication of SR in the effects of DEP associated or not with TLR agonists on monocyte-derived DC (MDDC). For this, we studied the regulation of CD36, CXCL16, LOX-1, SR-A1 and SR-B1 expression on MDDC treated with DEP associated or not with TLR2, 3 and 4 ligands. Then, the capacity of SR ligands (dextran sulfate and maleylated-ovalbumin) to block the effects of DEP on the function of lipopolysaccharide (LPS)-activated DC has been evaluated.

**Results:**

Our data demonstrate that TLR2 agonists mainly augmented CXCL16, LOX-1 and SR-B1 expression whereas DEP alone had only a weak effect. Interestingly, DEP modulated the action of TLR2 and TLR4 ligands on the expression of LOX-1 and SR-B1. Pretreatment with the SR ligand maleylated-ovalbumin but not dextran sulfate inhibited the endocytosis of DEP by MDDC. Moreover, this SR ligand blocked the effect by DEP at low dose (1 μg/ml) on MDDC phenotype (a decrease of CD86 and HLA-DR expression) and on the secretion of CXCL10, IL-12 and TNF-α. In contrast, the decrease of IL-12 and CXCL10 secretion and the generation of oxygen metabolite induced by DEP at 10 μg/ml was not affected by SR ligands

**Conclusion:**

Our results show for the first time that the modulation of DC functions by DEP implicates SR. TLR agonists upregulated SR expression in contrast to DEP. Interfering with the expression and/or the function of SR might be one way to limit the impact of DEP on lung immune response.

## Background

Airway mucosa represents the first line of defence against invading airborne pathogens and particulate matters. A high level of airborne particulate matters within the inspired air is associated with an increased incidence of respiratory diseases like allergic asthma and rhinitis [1]. Among these pollutants, road traffic and particularly the diesel cars represent a major source of particulate matters in urban area. Exposure to diesel exhaust particles (DEP) is associated with exacerbations of asthma, chronic obstructive pulmonary disease and allergic rhinitis [2-4].

DEP exert immunoregulatory functions through their first action on resident cells in the lung including macrophages, airway epithelial cells, and dendritic cells (DC). DC has been shown as playing a key role in the control of the lung immune response. These effects induced by DEP are probably responsible for its adjuvant activity that promotes pro-allergic sensitization to common environmental allergens [5], exacerbation of existing airway diseases [6,7], and increased susceptibility to respiratory virus infections like influenza [8] or RSV infections [9]. Regarding the modulation of DC functions, DEP do not induce their maturation but rather slightly modulate the response to potent maturation agents such as lipopolysaccharide (LPS), a ligand of Toll-Like Receptor (TLR)4 [10,11]. This effect involves the generation of reactive oxygen species (ROS) and the inhibition of NF-κB activation [12]. However, the early mechanisms by which DEP affect DC functions are not completely understood.

Whereas alveolar macrophages mainly reside in the alveolar region of the lung, immature myeloid DC (mDC) constitute a dense network in close proximity to airway epithelial cells [13]. Due to their role in the lung immune response, mDC are also determinant in the induction and the control of allergic asthma [14]. DC are professional antigen-presenting cells that are essential for initiating adaptive immune responses. They develop from bone marrow-derived CD34^+ ^precursor cells that travel in the bloodstream to secondary lymphoid tissues and mainly to the airway and gut mucosa. At a steady state and after exposure to danger signals, airway epithelial cells recruit immature DC or their precursors to sample inhaled antigens [15,16]. After antigen processing, maturing DC leave their resident sites towards the thoracic lymph nodes, where they efficiently prime naive T cells [17]. The T cell polarizing signals delivered by DC which are defined by the degree of cell maturation, determine the issue of the T cell response and the potential development of effector or suppressor T cells.

DC are able to discriminate unmodified self from non-self and altered/modified self thanks to a large family of receptors so called the pattern recognition receptors that include signalling receptors (e.g. Toll like Receptors (TLR)) and endocytic receptors including Scavenger Receptors (SR) [18,19]. The type of receptor involved in Ag capture will determine its processing and the issue of Ag presentation. Signalling and endocytic receptors cooperate to finely tune the degree of DC maturation and, by this way, to impact on T cell activation and polarization.

SR were originally identified by their ability to bind and internalize modified lipoproteins [18]. SR not only bind modified self such as oxidized LDL but also non self (microbes). In addition to their role in atherosclerosis, SR play critical roles in tissue homeostasis and innate immunity, e.g. by inducing apoptotic cell clearance. Different cell types express SR, such as endothelial cells, macrophages and DC. In comparison with macrophages, DC express a specific profile of SR belonging to different classes including SR-A1, MARCO (class A), SR-B1/CLA-1 and CD36 (class B), LOX-1 and CXCL-16 (also named SR-PSOX) (class D and E, respectively) [20]. The SR LOX-1, also known as OLR1 (oxidized low density lipoprotein (lectin-like) receptor 1) has also a C-type lectin-like domain (CTLD) of the type found in natural killer cell receptors (NKRs). CXCL16 also possesses a functional CXC chemokine domain active on T cells in addition to the mucin-like domain involved in the SR function. Although binding of DEP to alveolar macrophages was not inhibited by polyanionic ligand of SR [21], some SR such as MARCO are implicated in inert particle clearance [22].

These data suggest the implication of SR in the modulation of DC functions by DEP. Our aim is to demonstrate the involvement of SR in this process and the relationship with the activation by TLR ligands. Indeed TLR4 agonist is frequently associated with airborne particles [23]. In this work we first studied the effect of DEP on the SR expression in immature and mature DC, and second, the modulation by SR ligands of DEP uptake and effect. This role was evaluated in the context of an exposure to DEP alone or in association with TLR4 ligand. Our data demonstrate that DEP modulate the expression of some SR in immature and mature DC. Pretreatment with SR ligands allows to block some effects of DEP on cytokine production and costimulatory molecule expression by DC, at least in part through the modulation of DEP uptake. Taken together, our results show for the first time that the modulation of DC functions by DEP implicates the mobilization of SR.

## Results

### Modulation of mRNA expression of the SR CD36, CXCL16, LOX-1, SR-A1 and SR-B1/CLA-1 by DEP and TLR ligands

**Figure 1:**
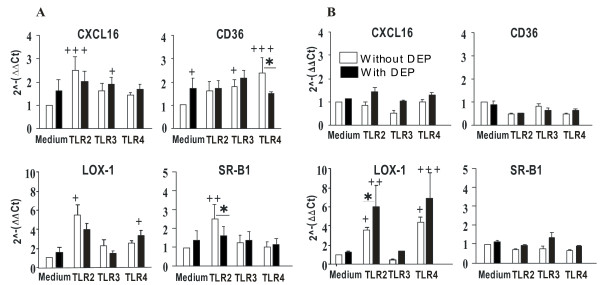
**DEP and PAMP modulate mRNA expression of Scavenger Receptors in MDDC**. MDDC were maintained in medium alone or activated with TLR2, -3 and -4 ligands (Pam_3_CSK_4 _(10 μg/ml), poly(I:C) (10 μg/ml) and LPS (1 μg/ml) respectively), associated or not with DEP (10 μg/ml) during 1 h (part A) and 3 h (part B). MDDC were harvested for mRNA isolation followed by measurement of CD36, CXCL16, LOX-1 and SR-B1 levels by quantitative RT-PCR. Results were expressed as the relative gene expression calculated for each experiment in folds (2^-(ΔΔCt)^) compared to unstimulated cells used as calibrator. Data reported the mean ± SEM from 4 independent experiments. +: p < 0.05; ++: p < 0.01; +++: p < 0.001 compared with cells in medium alone. ✻: p < 0,05 compared with TLR-treated cells.

**Figure 2:**
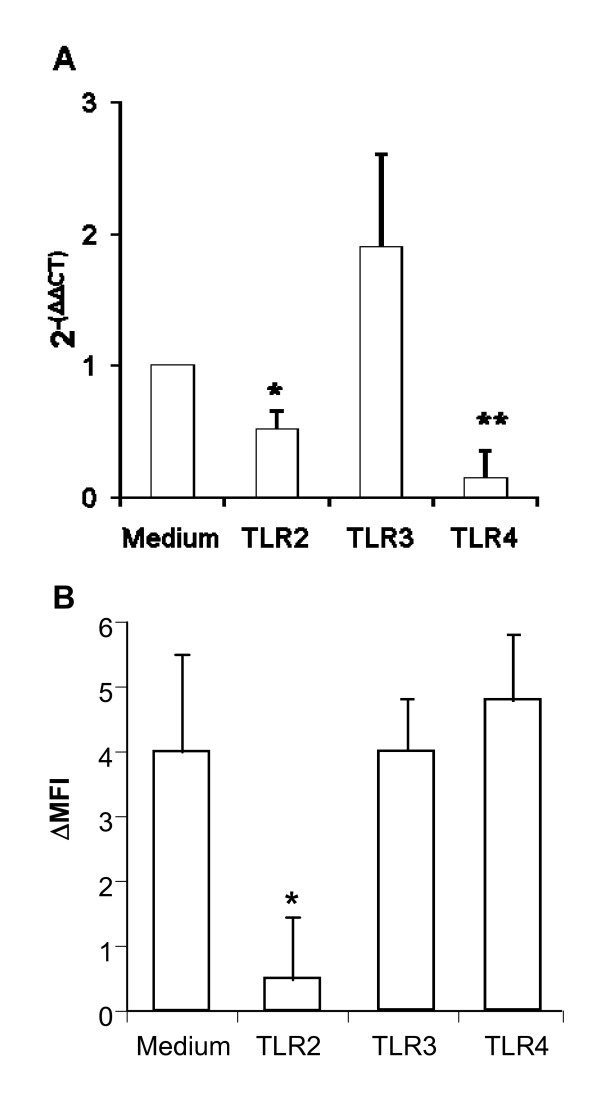
**TLR2 activation modulates protein expression of SR-A1 in MDDC**. MDDC were cultivated with TLR2, -3 and -4 ligands respectively Pam_3_CSK_4 _(10 μg/ml), poly(I:C) (10 μg/ml) and LPS (1 μg/ml) during 8 h for the mRNA expression (part A) and 24 h for the protein membrane expression (part B). A- MDDC were harvested for mRNA isolation followed by measurement of SR-A1 mRNA levels by quantitative RT-PCR. Results were expressed as the mean ± SEM of the relative gene expression calculated for each experiment in folds (2^-(ΔΔCt)^) compared to unstimulated cells used as calibrator (n = 5). B- Dendritic cells were labeled for SR-A1, and analyzed by flow cytometry. Data are expressed as the mean ± SEM from 5 independent experiments. *: p < 0.05; **: p < 0.01 compared with cells in medium alone.

We first determined whether DEP alone or in costimulation with TLR2 (Pam3CSK4 (10 μg/ml)), -3 (Polyinosinic-polycytidylic acid (poly(I:C))) (10 μg/ml), -4 (Lipopolysaccharide (LPS)) (1 μg/ml) ligands, modify the SR CD36, CXCL16, LOX-1, SR-A1 and SR-B1 mRNA expression in monocyte-derived DC (MDDC) using quantitative RT-PCR. Preliminary experiments showed that the optimal time of mRNA expression were 1 and 3 h stimulation (data not shown). DEP did not markedly increase the mRNA level of SR although a weak effect on CD36 was observed after 1 h stimulation (p < 0.05) (Fig 1A). The TLR2 ligand significantly increased the mRNA level of CXCL16 (p < 0.001) and SR-B1 (p < 0.001), after 1 h stimulation, and LOX-1 after 1 and 3 h stimulation (Fig 1A and 1B) (p < 0.05). TLR3 agonist slightly enhanced the mRNA level of CD36 (p < 0.05) after 1 h stimulation. TLR4 ligand significantly increased the mRNA expression of CD36 after 1 h stimulation (p < 0.001), and LOX-1 after 3 h stimulation. Expression of SR-A1 mRNA was not modulated by TLR agonists after 1 and 3 h stimulation in DC (data not shown). However, at 8 h activation, the TLR2 and TLR4 agonists significantly decreased its expression (p < 0.05, Fig 2A) whereas the poly(IC) had no activity.

Activation by DEP combined with TLR ligands resulted in the inhibition of TLR2 ligand-induced SR-B1 expression (p < 0.05), and TLR4 ligand-induced CD36 expression (p < 0.05) after 1 h stimulation. In contrast, DEP enhanced at 3 h the effect of TLR2 (p < 0.05) and TLR4 ligands on LOX-1. Exposure to DEP did not affect the expression of SR-A1 even in the presence of TLR agonists (data not shown).

Taken together, the results showed that DEP alone had a weak effect on SR expression in comparison with that of the TLR ligands. However, DEP modulated some of the stimulatory properties of TLR agonists.

### Modulation of protein expression for the CD36, CXCL16, LOX-1, SR-A1 and SR-B1/CLA-1 by DEP and TLR ligands

**Figure 3:**
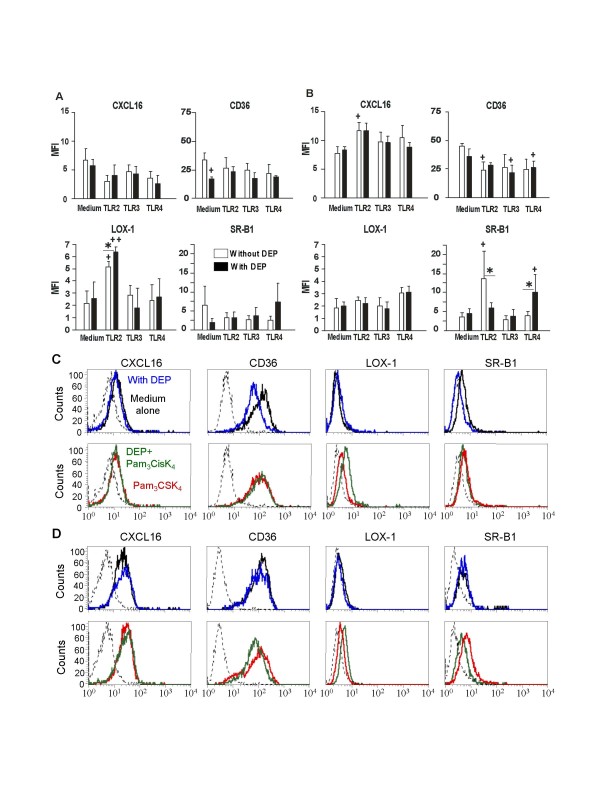
**DEP and PAMP modulate protein expression of Scavenger Receptors in MDDC**. MDDC were cultivated with TLR2, -3 and -4 ligands respectively Pam_3_CSK_4 _(10 μg/ml), poly(I:C) (10 μg/ml) and LPS (1 μg/ml), associated or not with DEP (10 μg/ml). A-B Dendritic cells activated during 6 h (A) and 24 h (B) were labelled for CD36, CXCL16, LOX-1 and SR-B1, and analyzed by flow cytometry. Data are expressed as the mean ± SEM from 3 to 7 independent experiments. +: p < 0.05; ++: p < 0.01 compared with unstimulated cells. ✻: p < 0,05 compared with TLR-treated cells. C-D Flow cytometry histograms of a representative experiment. MDDC were cultivated for 6 h (C) and 24 h (D) with DEP (blue line), with TLR2 ligand (Pam3CSK4, red line), and with both stimuli (green line) as compared with cells in medium alone (black line).

We next determined by flow cytometry the effect of these stimuli on SR membrane expression in MDDC activated during 6 and 24 h. At the opposite of the mRNA expression, DEP alone significantly decreased after 6 h stimulation the expression of CD36 (p < 0.05) and SR-B1 (p = NS) whereas it did not affect the level of CXCL16 and LOX-1 (Fig 3A and 3C). TLR2 ligand significantly increased the expression of LOX-1 after 6 h stimulation (p < 0.05) and CXCL16 and SR-B1 after 24 h stimulation (p < 0.05) whereas it significantly decreased the expression of CD36 after 24 h stimulation (p < 0.05) (Fig 3A–D). The activation by TLR3 agonist only tended to increase CXCL16 expression after 24 h. The TLR4 ligand increased LOX-1 after 24 h stimulation, whereas it significantly decreased the expression of CD36 after 24 h stimulation (p < 0.05). The effect of TLR2 agonist was illustrated in figure 3C–D by histograms of flow cytometry from a representative experiment. In contrast with the effect of LPS and poly(IC), the TLR2 agonist significantly decreased the expression of SR-A1 after 24 h activation (Fig 2B) but not after 6 h (data not shown).

Associated with TLR ligands, DEP decreased the effect of the TLR2 ligand on SR-B1 expression after 24 h stimulation (Fig 3B and 3D), but increased the action of the TLR4 ligand after 6 h stimulation (p < 0.05) (Fig 3B). Moreover, DEP significantly enhanced the action of TLR2 ligand on LOX-1 expression at 6 h stimulation (p < 0.05).

**Figure 4:**
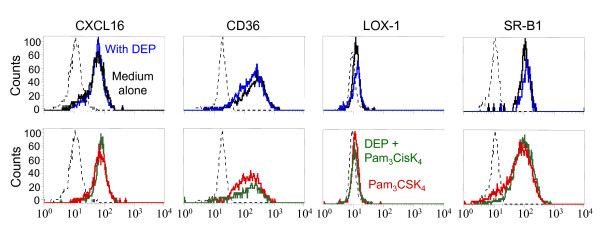
**DEP and TLR2 agonist modulate the total protein expression of CD36 and CXCL16 in MDDC**. MDDC were cultivated during 24 h with DEP (blue line), with TLR2 ligand (Pam_3_CSK_4_, red line), and with both stimuli (green line) as compared with cells in medium alone (black line). Dendritic cells were labelled after cell permeabilization for CD36, CXCL16, LOX-1, SR-B1 and with an isotype control (dotted line) and then, analyzed by flow cytometry.

Since SR had a rapid turnover, we have evaluated the total expression of SR in DC after 6 and 24 h incubation by immunofluorescence on permeabilized cells. Whereas no modulation was detected at 6 h, activation for 24 h by TLR2 and TLR4 ligands significantly inhibited (33% and 39% decrease, respectively) the intracellular labeling of anti-CD36 antibody as reported for TLR2 in the fig 4. DEP partially reversed the effect of TLR2 agonist whereas the particles alone did not modulate CD36 expression. No modulation was observed for SR-B1 and LOX-1. Concerning CXCL16, activation by TLR2 agonist increased the intracellular expression of this SR as detected on the membrane (22% increase, p < 0.05). In contrast, addition of DEP did not affect its expression.

Taken together, the results showed that, as reported for mRNA expression, activation by TLR ligands modulate SR membrane expression in different ways. DEP alone tend to decrease the expression of CD36 and SR-B1. Moreover, DEP modulated the effect of TLR2 and TLR4 ligands on the level of LOX-1 and SR-B1.

### Modulation by DEP of LPS-induced MDDC maturation: effect of SR ligands

Since DEP modulated the expression of HLA-DR, CD83 and CD86 in LPS-stimulated MDDC [10], we studied the capacity of poly-specific SR ligands (maleylated ovalbumin and dextran sulfate) to reverse the action of DEP on MDDC maturation.

**Figure 5:**
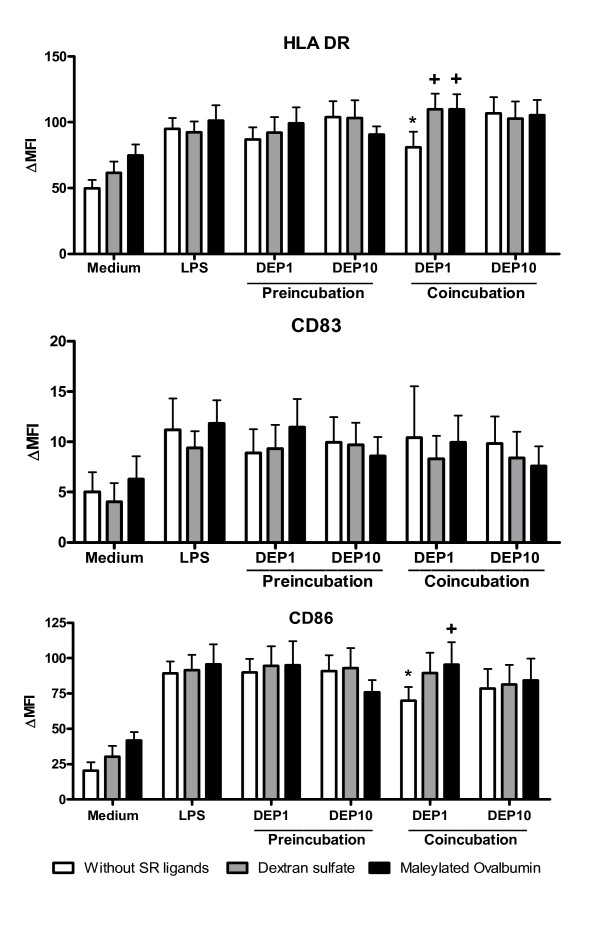
**DEP modulate LPS-induced MDDC maturation: effect of SR ligands**. MDDC were preincubated 2 h with 20 μg/ml dextran sulfate, 100 μg/ml maleylated ovalbumin associated or not with 1 or 10 μg/ml DEP and then, 100 ng/ml LPS were added (Preincubation). For the coincubation, the three ligands are added simultaneously. After 24 h incubation, MDDC were labeled for HLA-DR, CD83 and CD86 and were analyzed by flow cytometry. Data are expressed as the mean ± SEM from 7 independent experiments. ✻: p < 0.05 compared with LPS-treated cells. +: p < 0.05 compared with the condition without SR ligand.

As previously described, the effect of DEP was different according to the protocol (preincubation with DEP versus co-incubation with DEP and LPS) (Fig 5). DEP at the dose of 1 μg/ml significantly inhibited the LPS-induced upregulation of CD86 and HLA-DR (p < 0.05) when the ligands were added simultaneously. Addition of dextran sulfate and maleylated-ovalbumin blocked the inhibitory effect of DEP on LPS-stimulated DC (p < 0.05). In contrast, these SR ligands did not modulate the action of LPS alone on the 3 markers of maturation.

Preincubation with DEP at 1 μg/ml had a weak inhibitory effect on LPS-induced CD83 expression in comparison to LPS alone, whereas HLA-DR and CD86 were not modified. Addition of maleylated-ovalbumin reversed the action of DEP on CD83 expression. Whereas DC-SIGN expression was not affected by DEP treatment, these particles have an additive effect on the LPS-induced decrease of the Mannose Receptor expression (data not shown). Addition of SR ligands did not antagonize this activity (data not shown).

Taken together, these data confirmed that DEP modified the phenotype of LPS-induced matured MDDC, whereas SR ligands blocked the effects of DEP.

### Modulation by DEP of LPS-induced cytokine and chemokine production by MDDC: effect of SR ligands

**Figure 6:**
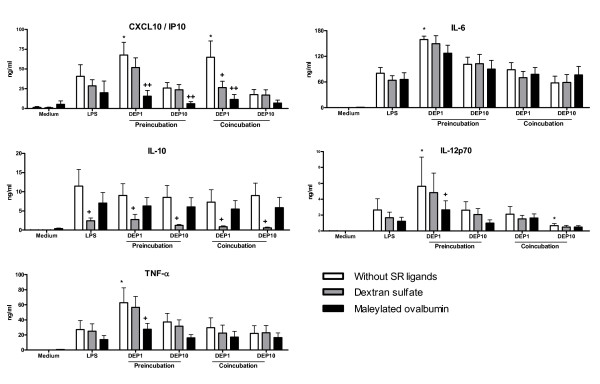
**DEP modulate LPS-induced cytokine and chemokine production by MDDC: effect of SR ligands**. MDDC were preincubated 2 h with 20 μg/ml dextran sulfate, 100 μg/ml maleylated ovalbumin associated or not with 1 or 10 μg/ml DEP and then, 100 ng/ml LPS were added (Preincubation). For the coincubation, the three ligands are added simultaneously. After 24 h incubation, the concentrations of IP-10, IL-12, IL-6 and TNF-α were measured by ELISA. Data are expressed as the mean ± SEM from 8 independent experiments. ✻: p < 0.05 compared with LPS-treated cells. +: p < 0.05; ++: p < 0.01 compared with the condition without SR ligand.

The involvement of SR in the effect of DEP on LPS-induced cytokine and chemokine secretion by MDDC was also studied. Activation by LPS alone increased the production of CXCL10/IP-10, IL-6, IL-10, IL-12p70 and TNF-α (Fig 6) whereas DEP alone had no effect (data not shown). Dextran sulfate (but not maleylated ovalbumin) strongly inhibited the LPS-induced production of IL-10 whereas addition of SR ligands did not significantly modulate the other cytokines.

Preincubation with low dose (1 μg/ml) of DEP significantly increased the LPS-induced production of CXCL10/IP-10, TNF-α, IL-6 and IL-12p70 (p < 0.05). Maleylated-ovalbumin strongly inhibited the production of CXCL10/IP-10 (p < 0.001), IL-12 (p < 0.05) and TNF-α (p < 0.05) induced by low dose of DEP, whereas dextran sulfate had no significant activity. Surprisingly, IL-6 secretion was not modified by treatment with SR ligands. Preincubation with high dose of DEP did not have any significant effect.

Coincubation with low dose of DEP significantly increased CXCL10 production induced by LPS (p < 0.05), a modulation which is antagonized by treatment with both SR ligands, dextran sulfate (p < 0.05) and maleylated ovalbumin (p < 0.001). In contrast, simultaneous addition of LPS and the highest dose (10 μg/ml) of DEP inhibited the production of CXCL10 and IL-12 in comparison with LPS alone. Nevertheless, the addition of SR ligands did not neutralize this effect. Preincubation and simultaneous treatment with DEP with and without maleylated-ovalbumin had any effect on IL-10 synthesis (Fig 6).

Taken together, these data showed that low dose of DEP increased the synthesis of cytokines by LPS-stimulated DC, whereas the exposure to high dose was able to inhibit CXCL10 and IL-12 production. Preincubation with maleylated-ovalbumin mainly blocked the effect of low dose of DEP but did not antagonize the activity of the high dose.

### Effect of DEP on DEP endocytosis and ROS production by DC

**Figure 7:**
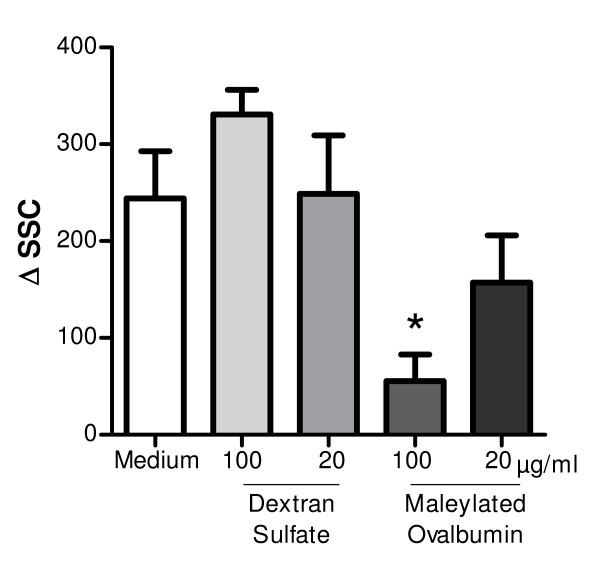
**SR ligands modulate DEP endocytosis**. MDDC were preincubated for 15 min with 20 and 100 μg/ml dextran sulfate or maleylated ovalbumin and then, DEP (10 μg/ml) was added. After 6 h incubation, side scatter (SSC) of MDDC was analyzed by flow cytometry. Data are expressed as the difference between the SSC in DEP-stimulated and inactivated cells. The data reported the mean ± SEM from 5 independent experiments. ✻: p < 0.05 compared with cells with DEP alone (medium).

In order to elucidate the action mechanism of SR ligands, endocytosis of DEP by MDDC has been evaluated. The uptake of particles induced an increase in the granularity of the cells, which led to a greater scattering of the laser light in flow cytometry [24]. This is confirmed by our data reported in figure 7 showing that exposure to DEP slightly but significantly (p < 0.05) increased side scatter (SC) in DC. Preincubation with maleylated-ovalbumin but not with dextran sulfate dose dependently inhibited the uptake of DEP (p < 0.05).

**Figure 8:**
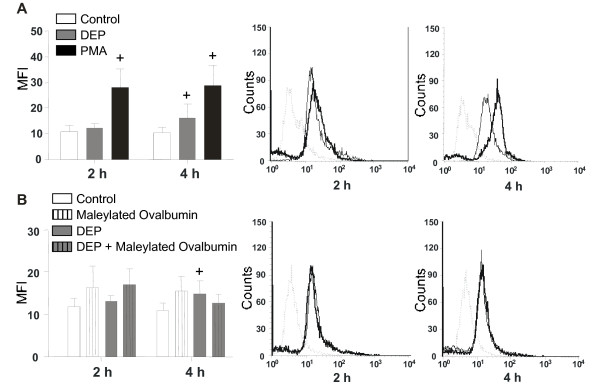
**Effect of DEP on ROS production by MDDC**. A. MDDC were incubated in medium alone, with DEP (10 μg/ml) or with PMA (100 ng/ml) for different time periods. ROS production was analyzed by flow cytometry. The left histogram reported the mean ± SEM from 6 independent experiments whereas the flow cytometry histograms show the data obtained in one representative experiment. +: p < 0.05 compared with cells in medium alone. B. Modulation by maleylated ovalbumin of DEP effect on MDDC activated for 2 and 4 h. Data in the left histogram are expressed as the mean ± SEM from 6 independent experiments. The flow cytometry histograms reported the data obtained in one representative experiment. +: p < 0.05 compared with cells in medium alone.

Since exposure to DEP secondarily induced generation of ROS involved in the modulation of DC functions [12], production of these metabolites by MDDC has been evaluated. At steady state, MDDC produced low level of ROS, as measured by flow cytometry. Activation of PMA significantly increased the generation of ROS which reached its maximum at 2 h (p < 0.05). DEP significantly increased ROS production after 4 h stimulation (p < 0.05), whereas this treatment had a weak effect at 2 h (fig 8A). On the left histogram, treatment with DEP (bold line) induced a strong gap at 4 h (about 40% of the ΔMFI obtained with PMA).

We also analyzed the capacity of maleylated-ovalbumin to modulate the DEP-induced ROS generation (Fig 8B). Maleylated-ovalbumin had no significant effect on DEP-induced ROS production observed after 4 h stimulation.

## Discussion

There is growing evidence that inhaled particulate matter derived from diesel contributes to the increased incidence of allergic diseases, respiratory infections and mortality [1,5-7,9]. The effect of DEP on lung immune response probably implicated the modulation of pulmonary DC functions.

In this study, we assessed whether DEP may affect the expression and the function of SR and by this way, can modulate the function of DC during activation by TLR ligands. Indeed, TLR ligands are frequently associated with airborne particles [23]. Here we showed that DEP modulate the activity of TLR ligands on SR expression in DC. Moreover, pretreatment with SR ligands blocks some effects of DEP on LPS-induced DC maturation and cytokine production through a still unknown mechanism.

Within airway mucosa, particulate matter-exposed bronchial epithelial cells secrete the DC chemoattractant CCL20/MIP-3α, CCL2/MCP-1, and CCL5/RANTES [25]. Therefore, one might expect that DC would be among the first cells to interact with inhaled particulate matter. In the current study, we used well-established protocols that are thought to yield immature MDDC representative of those present *in vivo *at mucosal sites [26]. Since respiratory tract DC are rapidly derived from circulating precursors [27,28], we believe our experiments provide a reasonable approximation of how DC and DEP interact *in vivo*. In addition, DC can insert dendrites between airway epithelial cells to directly capture the particle or microorganism within the airway lumen [29].

### Modulation of SR expression

There are few studies about the regulation of the expression of SR on DC. Concerning the regulation of class-A SR in DC, Amiel *et al *showed that SR-A expression is upregulated during DC maturation, and is correlated with the expression of the murine DC marker CD11c [30]. Another study showed that advanced glycosylation end (AGE)-BSA upregulated SR-A expression on DC via MAP kinases pathway (Jnk) [31]. The last work showed the upregulation of SR-B1 during the differentiation of MDDC. SR-B1 expression was suppressed by LPS, IFN-γ and TNF-α in monocytes and macrophages [32]. In the same way, we detect a transient decrease of SR-B1 expression in LPS-stimulated MDDC. We have also evaluated on MDDC the protein expression of MARCO, a SR involved in the particle clearance within the lung. Using a mAb (PLK1 clone; HBT, Uden, The Nederlands), our data revealed that this SR is nearly undetectable on MDDC by flow cytometry after intracellular and extracellular labeling, whatever the condition of stimulation (data not shown).

In this study, the selection of the five SR is based on their expression in airway mucosa particularly in airway epithelium and in MDDC (data not shown). In addition, these receptors are known to be implicated in the modulation of TLR activity [33-36]. Our results showed that DEP had a weak effect on the SR mRNA expression, in contrast with the strong effect induced by TLR2 and TLR4 ligands. However, DEP alone seems to decrease the protein expression of CD36 and SR-B1 suggesting their mobilization during their interaction with DEP. In contrast, TLR2 ligand and at a lower level, TLR4 agonist enhances both the mRNA and protein expression of LOX-1, CXCL16 and SR-B1. Concerning SR-A1 and CD36, there is a clear dissociation between the TLR-dependent modulation of mRNA (increased) and protein (decreased) expression suggesting that their membrane expression in DC are mainly controlled by post-transcriptional mechanisms as previously reported [20]. Measurement of intracellular expression revealed that the level of CD36 and CXCL16 is parallel in both extracellular and intracellular compartment whereas this is not true for LOX-1 and SR-B1. According to these data, the mechanisms controlling the membrane expression of LOX-1 and SR-B1 are probably very different from those of CD36 and CXCL16. Since TLR3 activation had a weak effect on SR level in contrast to TLR2 and TLR4, we can suspect that MyD88-dependent pathway mobilized by both TLR is involved in the modulation of SR expression. Associated with pathogen-associated molecular pattern (PAMP), DEP modulate the activity of TLR2 and TLR4 ligands according to the SR. As previously reported [10-12], DEP can modulate the signalling pathways activated by TLR and by this way, controlled the modulation of SR expression. In addition, we can suspect that DEP uptake by MDDC directly mobilizes some SR such as CD36 and SR-B1 and interferes with the activity of TLR agonists on SR expression.

### Regulation of DC maturation induced by TLR ligand

There are some controversial data in the literature concerning the impact of DEP exposure on DC maturation. Most studies show that DEP alone have no effect on DC maturation whereas it can act as an adjuvant in order to increase the response to an allergen or a TLR ligand [10]. In contrast, DEP can also inhibit some signals induced by TLR activation and deviate the phenotype of mature DC towards a pro-Th2 type. Indeed, DEP inhibit IL-12 mRNA and protein expression in DC, and decrease IFN-γ production by T lymphocytes cocultured with DEP-exposed DC [11]. DEP in mice inhibited DC maturation (IL-12 production and co-stimulatory molecule expression) induced by TLR2, 3, 4 and 9 ligands [12]. Finally, another report also suggests that direct exposure to airborne particulate matter from diesel vehicles increased the expression of MHC class II and costimulatory molecules and the production of TNF-α, IL-12p40, IL-6, and VEGF [2].

In our model, DEP alone have no effect on DC maturation, suggesting that their effect on MDDC is dependent upon their origin and their preparation (addition of surface activator or surfactant) as previously reported [37]. In our hands, DEP modulate the LPS-induced DC maturation according to the timing and to the dose. We show that the low dose of DEP increases the synthesis of cytokine by LPS-stimulated DC, whereas the high dose blocks IP-10 and IL-12 production. Moreover, SR ligands are able to reverse some effects of DEP. One hypothesis to explain these data might be that there is a competition for the uptake and internalization of DEP. Our results demonstrated that mOva but not dextran sulfate inhibited in a dose-dependent manner the uptake of DEP. This suggests that it is not the only mechanism involved in the activity of poly-specific SR ligands. The fact that SR agonists only block the action of low dose of DEP, can be explained by an insufficient molecular ratio between SR ligands and DEP in order to inhibit the effect of the high dose. Another explanation might be that, according to the dose, different receptors are implicated in the DEP effect. This hypothesis is likely since both doses of DEP have an opposite effect on cytokine secretion by DC. The implication of the xenobiotic sensor AhR has been previously mentioned [38]. Moreover, SR are also described as coreceptors for TLR, DEP can interfere with TLR-induced signalling through the mobilization of SR. For example, activation with the TLR2 ligand Kp-OmpA is dependent of both SREC-1 and LOX-1 or with diacylglycerides, of CD36 [34,39]. CD36, with established roles in recognition of endogenous and exogenous ligands, facilitates TLR2 signaling [40]. In addition, CXCL16 is involved in TLR9 activation by plasmacytoid DC [33]. Preincubation with maleylated-ovalbumin mainly block all the effects of low dose of DEP whereas dextran sulfate have a more restricted effect. Maleylated OVA binds to most of the SR, including the class II scavenger receptors SR-AI/II, SR-BI and CD36 [20]. Moreover, we have observed that CHO cells expressing either CXCL16, LOX-1 or SREC-1 strongly bind FITC-conjugated maleylated OVA (data not shown). Dextran sulfate has been reported to inhibit the binding of several ligands to SR-AI/II, SREC-I, CXCL16 and LOX-1 [41] whereas this ligand does not interact with CD36 [42]. Moreover, sulfated glyconconjugates, including dextran sulfate, share a binding inhibition pattern consistent with class A or C activity [43]. According to our data, we can suspect that class B SR (SR-B1, CD36) are presumably involved within DEP effect in DC. To confirm the implication of SR, it would be interesting to test other inhibitory methods like gene silencing.

The modulation of IL-12 and CXCL10 production (two major cytokines involved in the differentiation and the recruitment of Th1 cells) by SR ligands may have a strong impact on the capacity of DEP-exposed DC to polarize the T cell response. Since this effect is only observed at low doses, we can suspect that it is mainly involved in the long term effect of chronic exposure to these particles.

### DEP effect on DC phenotype

Our data demonstrate that coincubation with LPS and DEP affects the phenotype of mature DC, decreasing the expression of the costimulatory molecule CD86 and of the HLA-DR molecule. We demonstrate that this effect is dependent on SR mobilization. This process could impact the T cell response and polarization since CD86 is known to be implicated in Th2 cell development.

Recently, Porter *et al *demonstrate that airborne particulate matter from diesel vehicles enhanced the MR expression and potentialized antigen uptake (dextran-FITC) whereas LPS decreased both MR expression and antigen uptake [2]. In the present study, DEP alone had no effect on MR expression (data not shown) whereas it had an additive effect on the LPS-induced decrease of this receptor. As underlined above, this discrepancy is probably related to different origins of the particles.

### ROS production

It has been demonstrated in human and murine models that the effects of DEP on DC are associated with ROS production [11,12]. Notably, DEP-induced ROS production triggers the activation of a signaling pathway mediated by nuclear factor-erythroid 2 (NF-E2)-related factor 2 that suppresses IL-12 production [12]. In our model, the inhibition of IL-12 production as well as ROS production are only detected at high concentrations of DEP and both seem to be independent of SR mobilization. These data strongly suggest that the activation of NF-E2 in not dependent of SR-induced signalling pathways. At the opposite, SR seems to be involved in the upregulation of the cytokine production triggered by the low dose of DEP.

## Conclusion

Taken together, our results show that the modulation of DC functions by DEP involves the mobilization of SR. Moreover, the impact on DC functions appears to be different according to the dose and probably implicates different signalling pathways. Interfering with the expression and/or the function of SR might be one way to limit the impact of DEP on lung immune response and on the induction and the exacerbation of lung diseases.

## Methods

### Preparation of DEP

We used standard DEP (standard reference material (SRM) 2975) obtained from the National Institute for Standards and Technology (NIST, Gaithersburg, USA). The material was collected from a filtering system designed specifically for diesel-powered forklifts. Its chemical composition is mentioned in the Certificate of Analysis from NIST. DEP were diluted in a solution containig 0,04% Dipalmitoyl Phosphatidylcholin (Fluka Chemie, Buchs, Switzerland). To minimize aggregation, DEP were sonicated for 15 minutes and shaked prior to their dilution. The suspension was diluted in culture medium to the final concentrations required for exposure of the cells.

### Preparation of MDDC and DEP exposure

Blood monocytes from healthy volunteers were purified by positive selection over a MACS column using anti-CD14-monoclonal antibodies (mAb) conjugated microbeads (Miltenyi Biotec GmBH, Bergisch Gladbach, Germany) and were differentiated into dendritic cells by standard procedures [26]. Briefly, monocytes were cultivated at 1 × 10^6 ^cells/ml for 5 days in RPMI 1640 supplemented with 10% heat-inactivated FCS (Invitrogen, Paisley, UK) containing 10 ng/ml IL-4 and 25 ng/ml GM-CSF (PromoCell, Heidelberg, Germany).

At day 5, CD14^-^CD11c^+^HLA-DR^low ^immature monocyte-derived DC were obtained as characterized by their phenotype and the low level of cytokine production (data not shown).

Cells were either left untreated or were exposed for 1, 3, 6, 8 or 24 h to DEP (10 μg/ml) with or without the TLR2 ligand Pam3CSK4 (10 μg/ml), the TLR3 ligand poly(I:C) (10 μg/ml) or the TLR4 ligand LPS (1 μg/ml) (Invivogen, San Diego, CA) depending on the experiments. The vehicle for DEP containing 0.04% dipalmitoyl phosphatidylcholin was added in the control wells.

To evaluate their maturation in the presence of DEP, the activation protocol was as follows: MDDC were preincubated with the SR ligands Dextran sulfate (20 μg/ml) or maleylated- and deglycosylated ovalbumin (100 μg/ml) with or without DEP (1 or 10 μg/ml) during 2 h before addition of LPS (100 ng/ml) for 24 h. In some experiments, the SR ligands, DEP and LPS were added at the same time. The preparation of SR ligands are endotoxin-free, as measured by the limulus amebocyte assay (Lonza, Verviers, Belgium). As previously described, dextran sulfate particularly targets SREC-1 and CXCL16 whereas maleylated-ovalbumin binds to most of the SR [44-46]. Cell viability was evaluated in each condition of activation by trypan blue exclusion after 24 h incubation and no significant decrease was detected in the presence of each activator or their combination.

### Real Time quantitative PCR

Total RNA was isolated from DC exposed to DEP and/or to TLR ligands. After 1 or 3 h incubation, cells were washed in sterile cold PBS, lysated by using TRIzol reagent (Invitrogen) and RNA were isolated according to manufacturer's instructions. RNA concentration was determined by spectrophotometry and its quality was evaluated by electrophoresis through a 0.8% agarose gel visualized using Gelstar staining. Retro-transcription and Real-Time quantitative PCR were performed using SuperScript™ Platinum^® ^SYBR^® ^Green Two-Step qRT-PCR Kit with ROX (Invitrogen, Paisley, Scotland) according to manufacturer's instructions. Forty-five cycles of cDNA amplification were performed at 55°C (30s) after hybridation at 60°C (20s). In order to obtain a normalized target value, the house-keeping gene actin was used. Forward and reverse primers for CD36, CXCL16, Lox-1 and SR-B1 were designed as follows: CD36 (forward 5'-TGTCCGCGAAGAAGGTACAA, reverse 5'-TCACTTCCTGTGGATTTTGCAC); CXCL16 (forward 5'-GGCTTTGGACCCTTGTCTCTTG, reverse 5'-TTGCGCTCAAAGCAGTCCACT); LOX-1 (forward 5'-AGTGGACACAATTACGCCAGGT, reverse 5'-ATCTGCCCTTCCAGGATACGA); SR-A1 (forward 5'-TTCAAAGCTGCACTGATTGCC, reverse 5'-TTCTTCGTTTCCCACTTCAGGA); SR-B1 (forward 5'-TGACGATCCCTTCGTGCATT, reverse 5'-CATCCCAACAAACAGGCCAA); actin (forward 5'-TCCTCACCCTGAAGTACCCCA, reverse 5'-AGCCACACGCAGCTCATTGT).

Results were expressed mean +/- SEM of the relative gene expression calculated for each experiment in folds (2^-(ΔΔCt)^) compared to unstimulated cells used as calibrator.

#### Chemokine and cytokine measurements

The concentrations of cytokines and chemokines in the culture supernatants were determined by sandwich enzyme immunoassay as described by the manufacturer, R&D systems for CXCL10/IP-10, IL-6, and TNF-α or Diaclone (Besançon, France) for IL-12p70.

### Flow cytometry

After the recovery of MDDC supernatants, cells were incubated with PBS/EDTA (2 mM) and were detached by scraping. MDDC were centrifuged and resuspended in PBS containing 2% FCS. Cells were labeled (30 min, 4°C) with murine FITC-conjugated anti HLA-DR and DC-SIGN mAb, PE-conjugated anti CD80, CD83 and Mannose Receptor mAb, APC-conjugated anti CD86 and CD11c mAb or mouse IgG isotype controls conjugated with FITC, PE or APC (BD Pharmingen, except for CD83 from Beckman Coulter). Cells were washed with cold PBS and fixed with 1% paraformaldehyde in PBS.

To study SR expression, cells were labelled (30 min, 4°C) with mouse anti-LOX-1 (HBt), -SR-B1 (BD) and -CD36 (Labvision), goat anti-CXCL16 (R&D systems) antibodies or the relevant isotype control. Binding of unlabelled Ab or isotype control was detected by addition of FITC-conjugated anti-mouse or PE-conjugated anti-goat IgG antibodies (Invitrogen). In some experiments, the procedure was reproduced with cells previously fixed and permeabilized according to the procedure of the kit manufacturer (BD Biosciences). For SR-A1, cells were directly labelled with an FITC-conjugated anti-SR-A1 monoclonal antibody (R&D Systems). Then, cells were washed, fixed with paraformaldehyde in PBS and 10000 events were analyzed on a FACScalibur flow cytometer with CellQuest software (Becton Dickinson). Results are expressed as the difference between median fluorescence intensity (MFI) with specific antibody minus the isotype control MFI (ΔMFI).

### Analysis of oxidative metabolism

10 μM of H2DCFDA (Dihydro-DichloroFluorescein Diacetate) was added to 2 × 10^5 ^DC cultured in PBS for 30 minutes before stimulation with DEP (10 μg/ml) associated or not with SR ligand maleylated-ovalbumine, positive control PMA (100 ng/ml) for 2 and 4 h. ROS generation was quantified by Flow Cytometry. Results are expressed as the difference between median fluorescence intensity (MFI) with DCFDA minus the autofluorescence control MFI (ΔMFI).

### Statistical analysis

Results are expressed as the mean ± SEM. The statistical significance of the differences between experimental groups was calculated by ANOVA1 with a Bonferroni post test (GraphPad Prism 4 Software, San Diego, USA). Results with a *P *value of less than 0.05 were considered significant.

## Abbreviations

Ag: Antigen; DC: Dendritic Cell; DEP: Diesel Exhaust Particle; mDC: Myeloid Dendritic Cell; MDDC: Monocyte-Derived Dendritic Cell; MFI: Median of fluorescence intensity; mOVA: maleylated ovalbumin; mRNA: Messenger Ribonucleic Acid; LPS: Lipopolysaccharide; PAMP: Pathogen-Associated Molecular Pattern; PMA: Phorbol Myristate Acetate; ROS: Reactive Oxygen Species; RSV: Respiratory Syncytial Virus; SR: Scavenger Receptor; TLR: Toll-Like Receptor.

## Competing interests

The authors declare that they have no competing interests.

## Authors' contributions

TS had performed and analyzed most of the data, had designed the study and wrote the article with PG. DA had participated to the cell culture experiments, to the QRT-PCR experiments and to the collection of data; she had also contributed to the design of the study. BS, JP and DY had participated to the development of the methodologies regarding the expression of SR and to the revising of the article. They also prepared the SR ligands and evaluated their binding to CHO cells. PhL contributed to the design and conception of the study and to the revising of the article. PG had acted as the main contributor for the design and the conception of the study. He had also coordinated the study. All authors have read and approved the final manuscript.
